# Stabilization of cyclohexanone monooxygenase by computational and experimental library design

**DOI:** 10.1002/bit.27022

**Published:** 2019-06-24

**Authors:** Maximilian J. L. J. Fürst, Marjon Boonstra, Selle Bandstra, Marco W. Fraaije

**Affiliations:** ^1^ Molecular Enzymology Group University of Groningen Groningen The Netherlands

**Keywords:** Baeyer–Villiger monooxygenase, computational design, cyclohexanone, stabilization, thermostability

## Abstract

Enzymes often by far exceed the activity, selectivity, and sustainability achieved with chemical catalysts. One of the main reasons for the lack of biocatalysis in the chemical industry is the poor stability exhibited by many enzymes when exposed to process conditions. This dilemma is exemplified in the usually very temperature‐sensitive enzymes catalyzing the Baeyer–Villiger reaction, which display excellent stereo‐ and regioselectivity and offer a green alternative to the commonly used, explosive peracids. Here we describe a protein engineering approach applied to cyclohexanone monooxygenase from *Rhodococcus* sp. HI‐31, a substrate‐promiscuous enzyme that efficiently catalyzes the production of the nylon‐6 precursor ε‐caprolactone. We used a framework for rapid enzyme stabilization by computational libraries (FRESCO), which predicts protein‐stabilizing mutations. From 128 screened point mutants, approximately half had a stabilizing effect, albeit mostly to a small degree. To overcome incompatibility effects observed upon combining the best hits, an easy shuffled library design strategy was devised. The most stable and highly active mutant displayed an increase in unfolding temperature of 13°C and an approximately 33x increase in half‐life at 30°C. In contrast to the wild‐type enzyme, this thermostable 8x mutant is an attractive biocatalyst for biotechnological applications.

## Graphical abstract

This work describes the stabilization of an e‐caprolactone‐producing cyclohexanone monooxygenase through protein engineering. Applying a computational pipeline for predicting stabilizing point mutations and an innovative shuffled library strategy for their combination, the authors report an increase of 13 °C of the melting temperature of the notoriously unstable enzyme, while the half‐life improved 33‐fold.

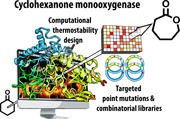

## 1 INTRODUCTION

The oxidation of ketones with peroxides and peracids is known as the Baeyer–Villiger (BV) reaction, named after the scientists who discovered it 120 years ago (Baeyer & Villiger, 1899). The credit for the “invention” goes to nature, however, where mechanistically analogously working enzymes called Baeyer–Villiger monooxygenases (BVMOs) were presumably already present in the last universal common ancestor (Mascotti, Lapadula, & Juri Ayub, 2015). BVMOs gained attention in the field of biocatalysis, because several representatives accept a very wide range of substrates, and catalysis often occurs chemo‐, regio‐, and stereoselectively (Leisch, Morley, & Lau, 2011). The BV reaction mechanism commences with a nucleophilic attack of the peroxide on the carbonyl carbon. In BVMOs, the catalytic entity is a peroxyflavin, generated from the reaction of molecular oxygen with reduced flavin adenine dinucleotide (FAD), which in turn received the electrons from NADPH. After the addition step, the so‐called “Criegee‐intermediate” rearranges, and generally, the carbonyl‐adjacent carbon that is better at stabilizing a positive charge migrates to the incorporated oxygen (Sheng, Ballou, & Massey, 2001). Enzymes can violate this “migratory aptitude” by binding the substrate such that the required antiperiplanar conformation of the canonically migrating bond is prevented, thus forcing the arrangement to the “abnormal” BV product (Li et al., 2018). An additional advantage of biocatalysis is the use of O_2_ as the oxidant, in contrast to the requirement of stoichiometric amounts of a peroxide reactant in the chemical reaction. This circumvents the peril of highly explosive concentrated peracids, as well as the generation of hazardous waste (Kamerbeek, Janssen, van Berkel, & Fraaije, 2003). Since these problems are above all relevant for industrial processes, biocatalytic alternatives should also be applicable at this scale.

One major hurdle to that end has efficiently been addressed in recent years: the prohibitively expensive nicotinamide cofactor consumed in the reaction is now routinely recycled by using whole‐cell systems, or by using a redox partner enzyme (Torres Pazmiño, Winkler, Glieder, & Fraaije, 2010). Various alternatives have been described, making for example use of dehydrogenases that regenerate NADPH from NADP^+^, either at the expense of a cheap sacrificial substrate, or with concurrent oxidation of a cascade reaction‐participating substrate (Bučko et al., 2016). Molecular fusions of the recycling enzymes facilitate handling and seldom affect enzyme activity (Aalbers & Fraaije, 2017; Mourelle‐Insua, Aalbers, Lavandera, Gotor‐Fernández, & Fraaije, 2019).

A remaining issue, however, is the poor stability of most BVMOs. In an industrial process, high temperatures and the use of cosolvents often are a prerequisite, or greatly increase efficiency or suitability. These conditions are incompatible with many BVMOs, which can inactivate within minutes, even in the buffer and at room temperature. The only truly thermostable BVMO, phenylacetone monooxygenase (PAMO) from *Thermobifida fusca*, unfortunately presents a strictly limited substrate scope and poor selectivities (Fraaije et al., 2004). PAMO was the first BVMO whose crystal structure was solved (Malito, Alfieri, Fraaije, & Mattevi, 2004), and despite intensive subsequent protein engineering (Bocola et al., 2005; Dudek et al., 2014; Parra, Acevedo, & Reetz, 2015; Reetz & Wu, 2009), catalytic efficiencies with most substrates remain too low to achieve industrially required productivities. One of the most important BV transformation, the conversion of cyclohexanone to ε‐caprolactone, a precursor to nylon‐6, is particularly difficult to achieve with PAMO. There is, however, a subgroup of BVMOs that is highly active on this substrate, the so‐called cyclohexanone monooxygenases (CHMOs). In fact, the first BVMO isolated was a CHMO stemming from *Acinetobacter* sp. NCIMB 9871 (AcCHMO). Although this enzyme is particularly unstable, it has become a prototype in BVMO research because of its high activities and substrate promiscuity, while often showing excellent selectivities.

A few studies have been reported in which AcCHMO's robustness could be improved: two studies introduced disulfide bridges (Schmidt, Genz, Balke, & Bornscheuer, 2015; van Beek, Wijma, Fromont, Janssen, & Fraaije, 2014), and the best mutants displayed an increase in the protein's melting temperature (*T*_m_), defined as the midpoint of the thermal denaturation curve, of 5 and 6°C, respectively. In an attempt to target the enzyme's oxidative stability, the third study introduced point mutations at sulfur‐containing residues (Opperman & Reetz, 2010). While not measuring *T*_m_, the authors reported on the temperature at which 50% of activity remained after incubation at various temperatures, which shifted by 6.8°C in the best mutant. When a recent study combined the most beneficial of these reported mutations, no *T*_m_ was reported, but it was found that none of thermostabilized mutants could outperform the wild‐type enzyme in terms of ε‐caprolactone production (Engel, Mthethwa, Opperman, & Kara, 2019). The authors attribute this result to the impaired catalytic parameters of the mutants. Although some of these studies have also quantitatively measured the mutants’ long‐term stability, a comparison of these results in terms of absolute values (Balke, Beier, & Bornscheuer, 2018) must be treated very cautiously: often, data is not available for the same temperature and as the assays are not standardized, the results may be influenced by variations in the particular measurement (Goncalves et al., 2017). Discrepancies across various studies, which can span over a few orders of magnitude, may furthermore be a result of the strong influence of additives on the stability of these enzymes (Goncalves et al., 2017). Regardless of the ultimate origin, it is clear that previous thermostability engineering efforts were only met with very limited success. A contributing factor to this outcome likely is the unavailability of a crystal structure of AcCHMO.

Recently, structures became available of two close homologs of AcCHMO: RhCHMO from *Rhodococcus* sp. HI‐31 (Mirza et al., 2009), and TmCHMO from *Thermocrispum municipale* (Romero, Castellanos, Mattevi, & Fraaije, 2016). The various structures of RhCHMO advanced mechanistical BVMO research, as it allowed intricate insight into both structural and catalytic features of CHMOs (Mirza et al., 2009; Yachnin et al., 2014; Yachnin, Sprules, McEvoy, Lau, & Berghuis, 2012). TmCHMO, on the other hand, can be seen as a big progression toward applicable CHMOs, and with a *T*_m_ of 48 °C, it is considerably more stable than both AcCHMO and RhCHMO (Romero et al., 2016). Yet, TmCHMO also shows a significantly lower activity on cyclohexanone, with a *k*_cat_ of 2 s^−1^ being approximately five times slower than that of the two homologs. Even lower activities are found for PockeMO, another recently described thermotolerant BVMO (Fürst et al., 2017). Because RhCHMO remains a prototype in BVMO research, and its substrate spectrum is highly similar to the by far best‐studied BVMO AcCHMO, we attempted to engineer RhCHMO toward increased thermostability. To that end, we applied our previously described framework for rapid enzyme stabilization by computational libraries (FRESCO; Wijma et al., 2014; Wijma, Fürst, & Janssen, 2018). This method uses two independent algorithms to calculate the difference in folding free energy, ΔΔG^Fold^, between a wild‐type structure and a single point mutant. After an in silico screening step supported by molecular dynamics simulations, selected mutants are expressed and tested for improved stability, and successful hits are combined to a final stabilized mutant. So far, we applied the FRESCO procedure to five enzymes, and achieved considerable improvements, with *T*_m_ increases up to 35°C (Arabnejad et al., 2017; Floor et al., 2014; Martin, Ovalle Maqueo, Wijma, & Fraaije, 2018; Wijma et al., 2014; Wu et al., 2016).

Here we describe the results gained by applying FRESCO to RhCHMO, where we could increase the *T*_m_ by up to 15°C in the most stable mutant. Although numerous stabilizing point mutations were encountered and confirmed the high success rate of the computational predictions, several complications needed to be overcome: many stability increases were small and not additive upon the combination, and some mutations caused insoluble or inactive enzymes. Besides investigating potential causes of these effects, we also devised a novel methodology to easily create random combinatorial mutant libraries, which aided in obtaining the most stable variant.

## 2 MATERIALS AND METHODS

### 2.1 General

All chemical reagents were purchased from Sigma‐Aldrich or TCI Europe, unless otherwise stated. Oligonucleotide primers were synthesized by Sigma‐Aldrich (Haverhill, UK) or Eurofins Scientific (Ebersberg, Germany). DNA sequencing was performed by GATC (Konstanz, Germany) and Eurofins Scientific.

### 2.2 Computational methods

The computational predictions by FRESCO were carried out by following the detailed protocol described in Wijma et al. (2018).

#### 2.2.1 Structure preparation

Using the YASARA software (Krieger & Vriend, 2014), the RhCHMO structure from PDB ID 4RG3 was prepared by adding hydrogens and removing alternative side chain configurations, buffer and active site ligand molecules. After using a YASARA script to identify residues with less than 5 Å distance from the active‐site ligand, the FAD and NADP^+^ cofactor, the remaining residues were subjected to Rosetta‐DDG (Kellogg, Leaver‐Fay, & Baker, 2011) and FoldX (Schymkowitz et al., 2005).

#### 2.2.2 Single mutant predictions

Rosetta's ddg_monomer version 2015.25.57927 was run using the “row 3 protocol” and these settings: ddg::weight_file soft_rep_design, ddg::iterations 50, ddg::local_opt_only true, ddg::min_cst false, ddg::mean true, ddg::min false, ddg::sc_min_only false, ddg::ramp_repulsive false, ddg::opt_radius 8.0, ddg::dump_pdbs false.

FoldX version 3.0b6 was used in the BuildModel mode with these settings: Temperature = 298 K, pH = 7, IonStrength = 0.05, moveNeighbours = true, VdWDesign = 2, numberOfRuns = 5.

#### 2.2.3 MD screening

The ΔΔG^Fold^ energy predictions of both programs were sorted and all mutations with a predicted energy of <−5 kJ mol^‐1^ were subjected to molecular dynamics (MD) simulations. For each mutant, five MD simulations with a random initial velocity seed were performed using a YASARA script employing Yamber3 as the force field. The system was warmed up from 0 to 298 K in 30 ps, followed by 20 ps equilibration, and a subsequent production run of 50 ps, where snapshots were sampled every 5 ps.

#### 2.2.4 Visual inspection

All mutants were visually inspected using YASARA with a custom plugin (Wijma et al., 2018). The average structure of each MD simulation was superimposed and the structure with the most notable differences eliminated, while the remaining four served as the basis for the visual screening of flexibility alteration. For experimental screening, only those were chosen, which showed no unfavorable hydrogen‐bonding alterations, no drastic hydrophobic surface exposure, and no increased local or overall flexibility. YASARA was also used to create the structural figures, except for Figure S10, which was created using the adaptive Poisson–Boltzmann solver (APBS) web server (Jurrus et al., 2018) with standard settings and visualizing the results using Chimera 1.10.2.

### 2.3 Molecular biology methods

The experimental procedure of screening targeted libraries was carried out by following the detailed protocol described in Fürst, Martin, Lončar, and Fraaije (2018).

#### 2.3.1 QuikChange mutagenesis

High throughput mutagenesis was conducted using a modified QuikChange protocol (Liu & Naismith, 2008) for which the primers were designed using AAscan (Sun et al., 2013). PCRs were conducted in 96‐well thin‐walled plates (Bio‐Rad) using the Pfu Ultra II Hotstart Master Mix (Agilent). As template served the native sequence of the *Rhodococcus* sp. HI‐31 gene (genbank accession number BAH56677) cloned into the previously described (Torres Pazmiño et al., 2009) pCRE vector, a pBAD‐NK derivative where the monooxygenase is N‐terminally fused to a 6×‐His‐tagged, thermostable phosphite dehydrogenase (PTDH). The PCR program was 95°C–3 min; (95°C–30 s; 55°C–30 s; 72°C–3 min) x 26; 72 °C–12 min. Subsequently, 10 U DpnI were added to each well to digest the template plasmid. After at least 4 hr reaction, 3.5 µl were transformed in chemically competent *E. coli* NEB10beta cells via 30 s heat shock at 42°C. Plating was conducted on 24‐well plates containing LB‐agar with ampicillin. A single colony was picked and grown on a 96‐well LB‐agar plate and sent for plasmid extraction and sequencing to Eurofins Genomics.

#### 2.3.2 Randomly shuffled library creation

The first library, which used the M4 mutant as a template, was based on the multichange isothermal mutagenesis (MISO) method (Mitchell et al., 2013), in which one Gibson‐assembles overlapping PCR fragments amplified using mutated primers. As seven mutations were to be randomized and two targeted the same residue (E91K and E91Q), our library size was 2^5^ × 3 = 96 possible combinations. We designed overlapping primers according to MISO, but to randomize, we performed the PCR with an oligonucleotide mix, combining a wild‐type sequence‐containing primer with a mutation‐containing primer, or two, in case of E91. We assembled the resulting fragment mix to full plasmids by Gibson cloning and analyzed 95 clones of the transformed library by DNA sequencing. The clones were mixed and each targeted position was either wild‐type or mutant, but in one case the ratio was strongly biased toward wild‐type (84%), and position 91 favored one of the mutations (60%; Figure S4A). This bias can originate from the PCR with mixed primers, where template‐binding may be altered by the mutation. Additionally, efficiency variations can occur in the assembly, where sequence‐dependent secondary structures impair fragment annealing. Although we attempted to mitigate these effects through silent mutations in the wild‐type primers and the use of secondary structure prediction software (Geneious), this precaution proved to be only partly successful.

For the second library, the M2 mutant served as a template, and mutations D10Y and D119R were excluded. To create the resulting library of again 96 possibilities without bias, we first created a plasmid with six mutations by MISO. Then, PCRs were performed with primers that generated fragments where the mutated site lies centrally. To randomize, we used a template mix combining the wild‐type plasmid and the 6x mutated plasmid (plus the single mutant plasmid with the third mutation for the fragment with position 91). With both the primer binding site and the assembly overlap now occurring at areas remote from the mutation, we expected to avoid bias. We sequenced 48 clones of the assembled and transformed library and performed a *χ* ^2^ test on the data (Microsoft Office Excel), using an equal 1:1 distribution as expected value range. We also sequenced a culture of a mix of all colonies by inoculating a single 5 ml culture tube with all of the 96 clones. The mixed signals in the electropherogram were analyzed using Geneious.

### 2.4 Biochemical methods

#### 2.4.1 Expression and cell‐free extract preparation

NEB10beta cells transformed with the pCRE‐RhCHMO construct were grown overnight in 200 µl LB_amp_ with vigorous shaking at 37°C in two identical 96‐well plate. Eight‐hundred microliters of TB_amp_ were added and the plates were shifted to 24°C with vigorous shaking. After approximately 36 hr, an OD_600_ of approximately 20 was reached and the cells were harvested by centrifugation. Cells were resuspended in 50 mM Tris/HCl pH 7.5 buffer containing 10 mM MgCl_2_, 0.5 mg/ml DNase I (NEB), and 1 mg/ml lysozyme (Sigma‐Aldrich). After 30 min incubation at 20°C, the cells were shock frozen in liquid N_2_ and thawed in a water bath. Cell debris was removed by centrifugation at 4°C and 2,250*g* for 45 min.

#### 2.4.2 Protein purification

The cell‐free extract (CFE) of the two identical plates was combined and subjected to affinity chromatography using 100 µl Ni^2+^‐Sepharose (GE Healthcare) in a 96‐well filter plate. The Ni^2+^ resin was allowed to incubate with the CFE for 1 hr, before removing the flow‐through by centrifugation. The resin was washed with 200 µl 50 mM Tris/HCl pH 7.5 and the same buffer with additionally 5 mM imidazole added. The pure protein was then eluted by adding 100 µl of 50 mM Tris/HCl pH 7.5 + 500 mM imidazole. Desalting was performed by using a PD MultiTrap G‐25 96‐well desalting plate (GE Healthcare) according to manufacturer's instructions.

#### 2.4.3 Thermostability screen

The purified protein's *T*_m_ was determined by the ThermoFAD method (Forneris, Orru, Bonivento, Chiarelli, & Mattevi, 2009). Twenty microliters of the desalted protein solution was transferred to a qPCR plate (iQ, Bio‐Rad) and subjected to a melt curve program in a qPCR machine (Touch Thermal Cycler; Bio‐Rad), which ramped the temperature from 20 to 99°C in 0.5°C increments after 10 s incubation and a fluorescent read step. For excitation, a 450 to 490 nm filter was used and for emission, a 515 to 530 nm filter.

#### 2.4.4 Enzyme kinetics

To determine the kinetic constants, reactions of RhCHMO with cyclohexanone and NADPH were followed spectrophotometrically (V‐660; Jasco) at 340 nm (ɛ_340_ = 6.22 mM^‐1^ cm^‐1^). The 200 µl reaction mixture contained 50 mM Tris/HCl pH 7.5, 0.05 µM RhCHMO, 100 µM NADPH, and 0 to 500 µM substrate. The mix was quickly transferred to a 0.1 ml quartz cuvette after addition of NADPH and the absorbance at 340 nm was measured for 45 s. The kinetic constants were calculated by fitting the data to the Michaelis‐Menten equation using the GraphPad Prism 6.

#### 2.4.5 Temperature optimum

The temperature optimum was determined by following the same procedure as for the enzyme kinetics determination, but using always 100 µM of cyclohexanone and varying the temperature. One hundred and eighty microliters of the enzyme‐substrate mix was incubated at the specified temperature for 2 min, to allow the solution's temperature to equilibrate. Addition of 20 µl NADPH then started the reaction, and the mix was transferred to a 0.1 ml quartz cuvette, which was placed in the preheated sample holder of the spectrophotometer.

#### 2.4.6 Stability over time

The enzyme was incubated at the specified temperature, after which a sample was taken and the residual activity determined by following the same procedure as specified above.

## 3 RESULTS AND DISCUSSION

### 3.1 In silico predictions

There are five different crystal structures of RhCHMO in the protein data bank (PDB), all of which have been suggested to represent a distinct conformation adopted by the enzyme throughout its catalytic mechanism. Although all structures contain both the FAD and the NADP cofactor, previous computational analysis of RhCHMO was based on the “closed” structure (PDB ID 3GWD), because it was assumed to represent the catalytically more relevant conformation in which the substrate is converted (Polyak, Reetz, & Thiel, 2012). While the “open” structure (3GWF) was proposed to represent the enzyme in the ligand‐free form, the lack of electron density for a large loop on the protein's surface already impedes a reliable computational analysis (Mirza et al., 2009). This so‐called control‐loop, stretching from residue 488 to 504, is also lacking in the “rotated” (3UCL; Yachnin et al., 2012) and “loose” (4RG4; Yachnin et al., 2014) conformations.

For the FRESCO calculations, we used the fifth, “tight” RhCHMO structure (4RG3; Yachnin et al., 2014), which presents a “closed” conformation with an ordered control‐loop, contains a ligand in the active site, and is the structure with the highest resolution. We applied the in silico protocols as previously described in detail (Wijma et al., 2018), which predict stabilizing mutations by applying the Rosetta‐DDG (Kellogg et al., 2011) and FoldX (Guerois, Nielsen, & Serrano, 2002) algorithms in combination with MD simulations. Instead of performing the calculations on all of the 10,260 possible point mutants (19 × 540 residues), mutagenesis of catalytically relevant positions was avoided by excluding residues in a distance smaller than 5 Å from the active site. As BVMOs stably bind to FAD and NADP^+^ (Sheng et al., 2001), we also had to exclude residues in the vicinity of these two cofactors (Figure S1). Although the preservation of cofactor affinity is an obvious aim, the main reason is that the Rosetta and FoldX algorithms are not parameterized to work with cofactors. Any calculations for these residues would, therefore, be unreliable. After ranking the calculations of the remaining 8,350 mutations by energy (Figure S1), the 853 variants that showed a ΔΔG^Fold^ ≤ −5 kJ mol^−1^ underwent short MD simulations, which included the cofactors. The generated in silico structures were then visually inspected. After discarding chemically unreasonable mutations and those where MD simulations suggested an increase in flexibility of the protein, we chose initially 114 point mutations for experimental verification (Figures S1, 1).

### 3.2 Stability determination of point mutants

We performed the experimental stabilization procedure (see Figure S1 for an overview of the workflow) from DNA mutagenesis to *T*_m_ measurements of purified enzymes following a protocol recently published as a step‐by‐step description (Fürst et al., 2018). A 96‐well format was maintained throughout nearly the entire procedure. First, we exchanged the targeted codon using the QuikChange method on a plasmid harboring the RhCHMO gene fused to an NADPH‐recycling PTDH‐encoding gene. After transformation into *E. coli* NEB 10 beta cells, we expressed the proteins on a 2 ml scale. The CFE of the lysed cells was then subjected to affinity chromatography that took advantage of an N‐terminal 6×‐histidine tag. The *T*_m_ of the purified protein was determined in a flavin‐fluorescence‐exploiting thermal shift assay called ThermoFAD (Forneris et al., 2009). As it is convenient to avoid full purification and perform *T*_m_ determinations using CFEs, but the results may vary significantly, we measured *T*_m_s for both the CFE and the purified proteins. With a coefficient of determination *R* ^2^ of only 0.68 (Figure S2), we deemed the *T*_m_ of RhCHMO to apparently be too strongly influenced by the soluble cellular components in the CFE and decided for *T*_m_ measurements to rely on purified proteins only.

**Figure 1:**
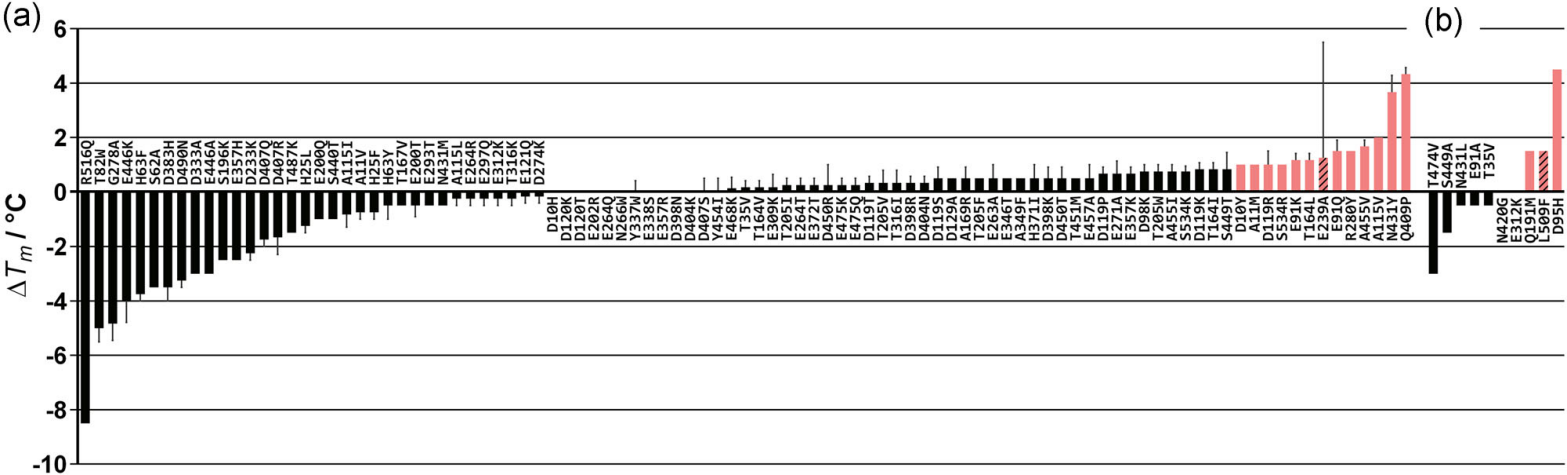
*T*_m_ increase of RhCHMO for the first (a) and second (b) set of point mutants. Stabilizing mutations with a *T*_m_ increase of at least 1°C are shaded pink, stabilizing mutations not considered for mutant combinations are indicated by a striped pattern. RhCHMO, cyclohexanone monooxygenase from *Rhodococcus* sp. HI‐31 [Color figure can be viewed at wileyonlinelibrary.com]

Out of 114 of the RhCHMO single mutants initially selected from the FRESCO calculations, 18 repeatedly gave no detectable ThermoFAD signal, likely caused by insufficient expression of soluble protein and/or a lack of FAD incorporation. Although BVMOs with lowered cofactor affinity can be reconstituted to the holoenzyme by adding an excess of FAD during purification, this is an unattractive feature for application and no reconstitution attempt was made. Of the remaining 96 mutants, 34 had a decreasing effect, 13 had no effect and 50 had an increasing effect on *T*_m_ (Figure 1a). Although this success rate compares favorably with previous FRESCO stabilizations, where typically 20 to 40% were found stabilizing (Arabnejad et al., 2017; Floor et al., 2014; Wijma et al., 2014), most of the *T*_m_ improvements we found for RhCHMO were very small. When we selected 13 mutations with a Δ*T*_m_ of at least 1°C and produced the proteins on a larger scale for verification, we were able to confirm the stabilizing effect for all but one mutation (E293A). The remaining 12 were subjected to the next step in the FRESCO procedure, the combination of mutations in a multiple mutant.

### 3.3 Mutant combination libraries

Initially, we combined the most stabilizing mutations by successive rounds of QuikChange. When we tested the mutant proteins, however, we noticed that the *T*_m_ increases were not additive and that the addition of one mutation (A11M) caused expression of an insoluble protein. The most stable mutant with a *T*_m_ increase of 6.3°C was the 4x mutant Q409P/N431Y/A115V/A455V (M4). As we also observed unsatisfactory results when adding mutations in another order, we decided to change strategy and devised a more systematic method.

We previously created a combinatorial library of wild‐type and mutant residues by golden gate shuffling using synthetic genes (Martin et al., 2018). To explore a method that avoids DNA synthesis, we now applied a PCR‐ and Gibson assembly‐based method. We decided to use the M4 mutant as a starting point and to exclude the A11M mutation, while randomizing the remaining seven mutations. As two mutations targeted position E91, the library size was 96 (2^5^ × 3). Initially, we created the library using an altered method described for the simultaneous mutation of several positions on a plasmid (Mitchell et al., 2013; Figure S3). Details of our adapted procedure, which is based on the direct generation of mixed wild‐type and mutated PCR fragments and Gibson assembly, are described in the experimental section. DNA sequencing of 95 clones of the library confirmed a shuffled library but revealed an uneven distribution of wild‐type and mutant in some cases (Figure S4A). When we expressed three 96‐well plates of individual clones (to achieve ~95% library coverage if there was no bias) and measured the purified protein's *T*_m_, we found only a few hits with marginally increased stability (data not shown). This seemed to suggest that our starting point was a dead‐end combination that had reached a thermostability plateau. Noticing also the absence of a signal in approximately 70% of the clones, we cross‐referenced the results with their sequences. We found that the mutants giving a signal always had the wild‐type residue at two positions (D10Y and D119R), which we thus deemed as incompatible for combinations (Figure S4B).

**Figure 2:**
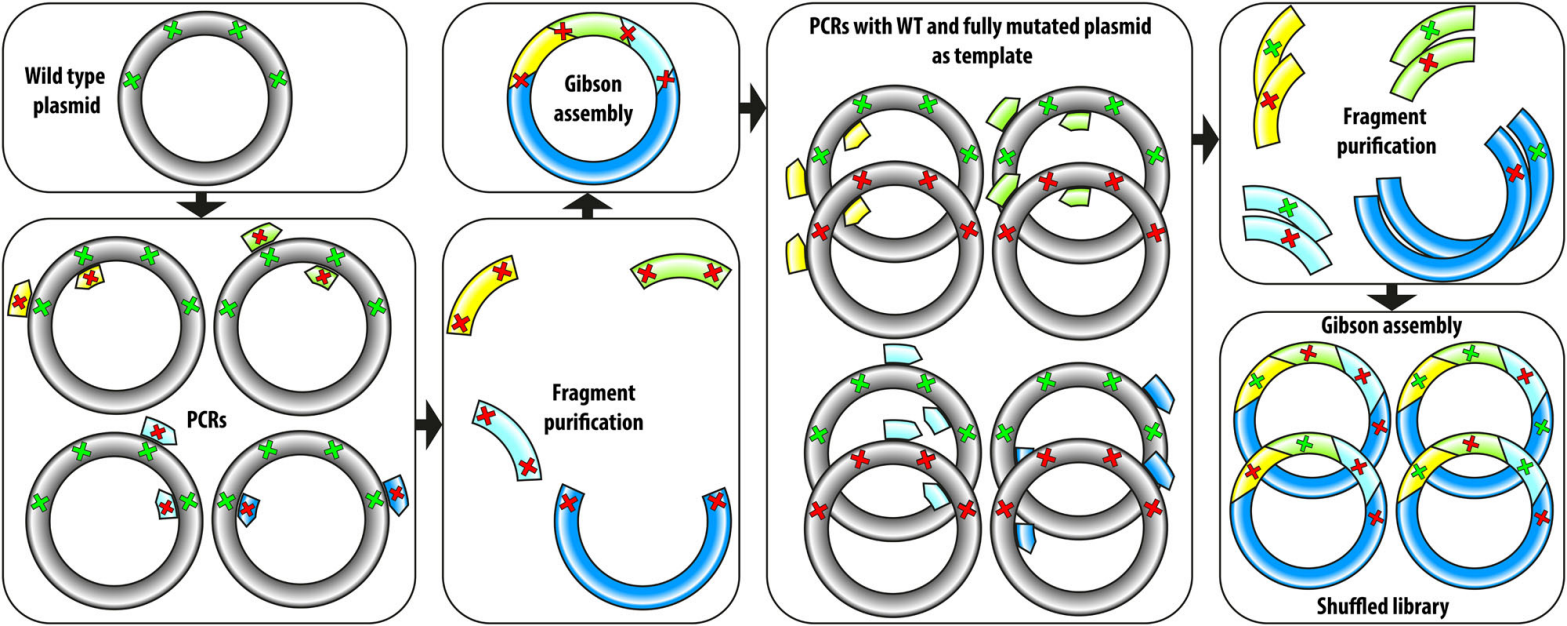
Method to create the second shuffled library without bias. The wild‐type plasmid was used as a template in PCRs with mutated primers to generate DNA fragments containing two mutations on each end. The purified fragments were assembled by Gibson cloning to obtain a plasmid with all mutations. An equal mix of this mutated and the wild‐type plasmid was used in the next round of PCR where primers were used that bound outside the mutated region to generate a mix of fragments with and without mutations. Upon Gibson assembly of this mix, the shuffled library was obtained. PCR, polymerase chain reaction [Color figure can be viewed at wileyonlinelibrary.com]

We decided to create a second library without these mutations and tried to escape the stability dead‐end by randomizing A115V and A455V, using the double mutant Q409P/N431Y (M2) as the new template. To furthermore create the resulting library of again 96 possibilities without bias, we also adapted the method for generating the library (Figure 2). We now first created a fully mutated plasmid and then generated mixed wild‐type and mutated PCR fragments with primers remote from the mutated site. We sequenced 48 clones of the assembled and transformed library and again found successful randomization. Furthermore, a *χ* ^2^ test on the data found no significant deviation from the expected 50% (or 33% in the case of position 91) ratio (Figure S5; Table S1). We also sequenced a culture of a mix of all colonies, and as the mixed signals in the electropherogram reflected the same distribution, we conclude that this suffices in verifying such libraries quickly (Figure S6).

**Figure 3:**
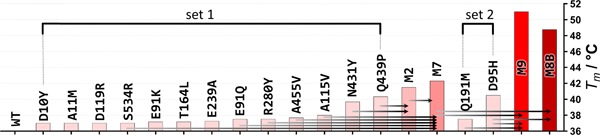
*T*_m_ increase of RhCHMO by a combination of mutations to a highly stabilized multiple mutant. RhCHMO, cyclohexanone monooxygenase from *Rhodococcus* sp HI‐31 [Color figure can be viewed at wileyonlinelibrary.com]

We then produced the proteins from three 96‐well plates of individual clones and measured *T*_m_. Although we found a much higher signal rate (81% of all clones), again no combination of mutations increased the thermostability of RhCHMO to a large extent (data not shown). At this point, the strongest increase in *T*_m_ with respect to the wild‐type was 7.3°C found for a 7x mutant (M7), only 1°C more stable than the M4 mutant (Figure 3).

### 3.4 Final stabilization mutations

Acknowledging the fact that we seemed to have found the largest possible *T*_m_ increase achievable with our initially selected mutations but being unsatisfied with the result, we decided to test an additional small set of point mutations. Revisiting the computationally predicted list of single mutants, we, therefore, selected 14 more mutations to be tested experimentally. Following the same procedure as before, we found no signal for four, a destabilizing or no effect for five, and a significant increase in *T*_m_ for three mutations (Figure 1b). At this point, an independent study found indications for an implication of one of the stabilizing residues, located in the substrate tunnel, on the substrate scope of a related CHMO (Fürst, Romero et al., 2018). For this reason, we excluded this mutation (L509F) from further combination. The other two mutations were directly added to our most stable mutant M7. Gratifyingly, we found a larger than additive effect on *T*_m_: while the single mutants, D95H and Q191M increased the *T*_m_ by 4.5 and 1.5°C, respectively, addition to M7 increased the *T*_m_ by 6.4 and 3.2 °C, respectively. When we added both mutations, the effect remained additive in the resulting mutant M9, which showed a *T*_m_ increase of 15°C with respect to wild‐type.

**Figure 4:**
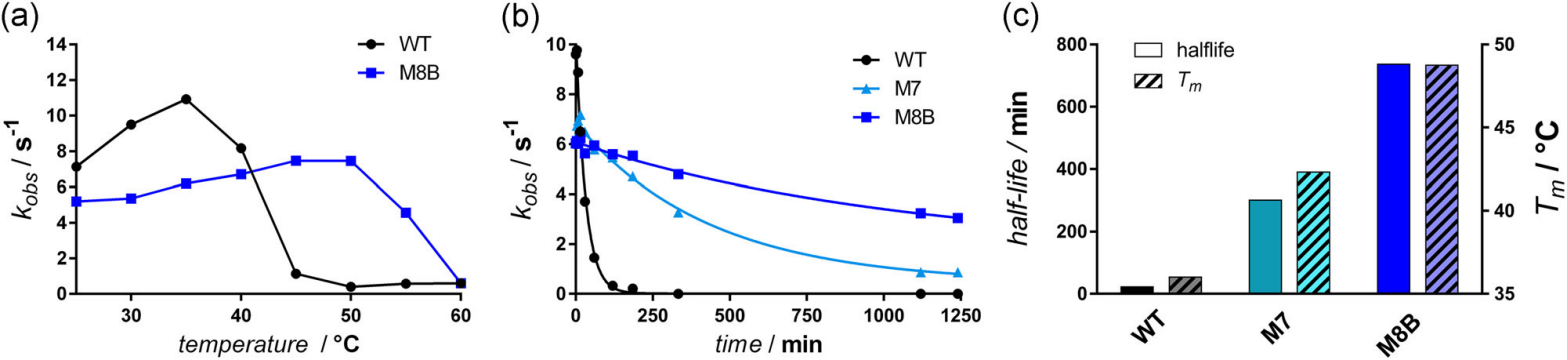
Comparison of the stabilized mutants with wild‐type RhCHMO. (a) Temperature optimum of wild‐type RhCHMO and M8B mutant. (b) Activity over time upon incubation at 30°C. (c) Comparison of *T*_m_ and half‐life. RhCHMO, cyclohexanone monooxygenase from *Rhodococcus* sp. [Color figure can be viewed at wileyonlinelibrary.com]

As the FRESCO protocol foresees to exclude residues in a 5 Å radius from the active site, changes in activity are rarely observed in stabilized mutants, with the exception of a common upshift of the temperature optimum. There are indications, however, that the activity of BVMOs is not purely determined by active‐site residues (Fürst, Romero et al., 2018). When we checked the most stable mutants for activity on the native cyclohexanone substrate, we found a highly diminished activity for the M9 mutant, but not for M7. We then looked at the activity of the 8x mutant with the second highest *T*_m_ (49°C), and found this mutant (M8B) to be highly active again. In comparison to M9, M8B only lacks mutation Q191M, which was thus identified as the activity‐abolishing mutation. We also determined the temperature optimum of the two enzyme variants and found a shift that was very much in line with their melting temperatures (Figure 4a). The kinetic constants were then measured at standard conditions, and we found a *k*_cat_ of 4.9 s^−1^ for the mutant compared to 9.2 s^−1^ for the wild‐type (Figure S7; Table 1). The high affinity for cyclohexanone was retained in the mutant, and the *K*_m_ could only be estimated to be <1 µM for both wild‐type and mutant (Table 1). To estimate the monooxygenases‐inherent degree to which NADPH oxidation is uncoupled from substrate oxygenation, we also measured rates in the absence of substrate. We found a very low uncoupling rate of 0.05 s^−1^ in the wild‐type, which only slightly increased in the mutant (0.13 s^−1^).

**Table 1:** Kinetic and stability properties of CHMOs compared

CHMO variant	*T*_m_	*k*_cat_	*K*_m_	Reference
AcCHMO	37.0°C	6 s^−1^	<4 µM	Romero et al. (2016)
TmCHMO	48.0°C	2 s^−1^	<1 µM	Romero et al. (2016)
RhCHMO	36.0°C	9.2 s^−1^	<1 µM	This study
RhCHMO M8B	48.8°C	4.9 s^−1^	<1 µM	This study

Abbreviation: CHMOs, cyclohexanone monooxygenases.

Previous FRESCO projects also demonstrated that the proteins’ *T*_m_ increase was accompanied by long‐term stability, that is longer retention of activity over time. To determine this characteristic for our most stable RhCHMO mutants as well, we measured the residual activity of the wild‐type, the M8B, and the intermediate M7 mutant after incubation at 30 and 37 °C (Figure 4b; Figure S8). These measurements demonstrated a strong correlation between *T*_m_ and stability over time, as the enzyme's half‐life of activity at 30°C was improved 33‐fold from 22 min in the wild‐type to 12.3 hr. For the M7 mutant, we measured a half‐life of approximately 5 hr, which lies approximately in the middle between wild‐type and M8B mutant, as does the *T*_m_ of this variant (Figure 4c). Similarly, the half‐life at 37°C increased from 8.6 min in the wild‐type to 135 min in the M8B mutant (Figure S8). We thus again demonstrated the high correlation between a protein's unfolding temperature and its stability over time. Considering the largely retained catalytic properties (Figure S7) and the high increase in stability over time, the RhCHMO M8B mutant is a much more suitable biocatalyst for application than both its wild‐type template and the very similar AcCHMO (Table 1).

### 3.5 Structural analysis of mutations

To rationalize some of the effects observed by the mutations, we analyzed the location and environment of the respective residues in the protein's structure.

**Figure 5:**
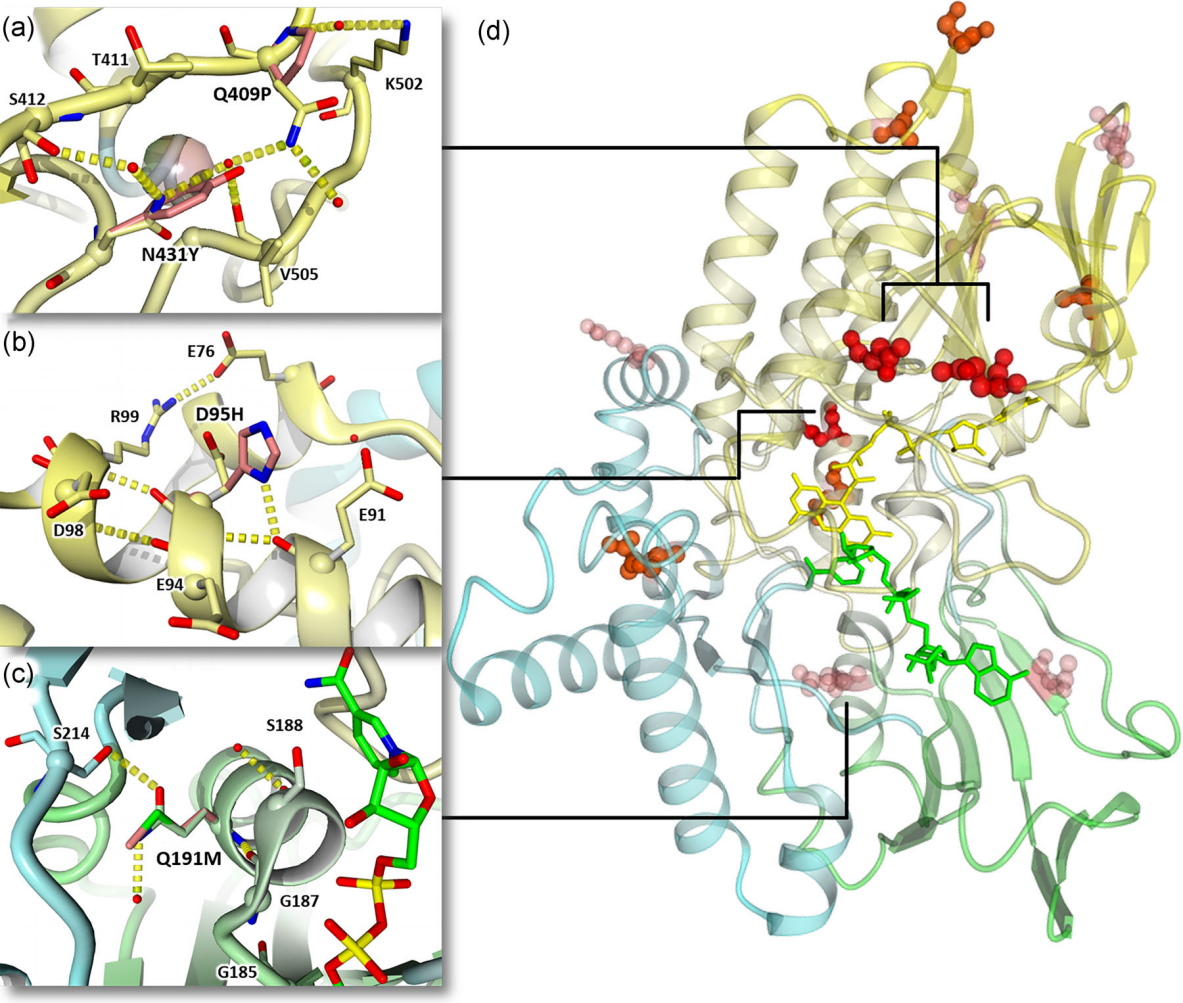
Mutations introduced in RhCHMO to increase thermostability. The crystal structure of RhCHMO (PDB ID 4RG3) shown in cartoon representation and colored yellow, green and cyan for FAD, NADP, and helical domain, respectively. FAD is shown in yellow and NADP^+^ as green sticks. (a–c) show the close up view of important residues, where the computationally predicted mutations are superimposed and shown as sticks with pink carbons. Alpha carbons are indicated by balls, hydrogen bonds as yellow dashed lines, and water molecules from the crystal structures as red balls. In (a) a transparent sphere indicates a cavity observed in the wild‐type, which is filled by the tyrosine mutation. (d) shows the overall protein structure and all mutations experimentally shown to be stabilizing are shown as balls in shades of red indicating the degree of stabilization. FAD, flavin adenine dinucleotide; RhCHMO, cyclohexanone monooxygenase from *Rhodococcus* sp HI‐31 [Color figure can be viewed at http://wileyonlinelibrary.com]

The two most stabilizing mutations of the first set, combined in the M2 mutant, were N431Y and Q409P, which are located very closely together (Figure 5a). For the N431Y mutation, a combination of improved protein packing and hydrogen‐bonding network seems to occur: in the wild‐type structure, the asparagine fringes a cavity, which is filled by the bulky tyrosine in the mutant. Furthermore, the carbonyl of wild‐type N431 is unable to hydrogen bond and this is prevented in the mutant, where the van der Waals interactions with the nearby V505 occur instead. The asparagine's amine group coordinates two water molecules, one of which is replaced by the tyrosine's hydroxyl group in the mutant, and which constitutes the link to Q409. For this residue, the effect of the mutation stems from elsewhere: Q409 is located on a surface‐exposed loop and, unlike proline, can hydrogen bond to the solvent waters. Analysis of the MD simulations indicates that the more rigid torsion angles forced upon the backbone by proline decrease the flexibility of the loop and nearby regions, which likely is the cause of the improved stability (Figure S9).

D95 is located at the center of a negatively charged surface patch (Figure S10A; Figure 5B). As no obvious hydrogen‐bonding impairments or flexibility alterations are observed in the histidine mutant, the stabilizing effect might be related to the charge change. One possibility is that the mutated residue forms a salt bridge, assuming that the solvent‐exposed histidine surrounded by acidic residues is mostly in the protonated form. Such an interaction is, however, not observed in the predicted structure. The stabilizing effect could therefore simply result from preventing a thermodynamically unfavorable clustering of anionic residues (Figure S10).

The activity‐abolishing effect of mutation Q191M could be related to a hydrogen bond that this residue establishes with a strictly conserved glycine that is part of the NADP binding Rossmann fold motif (Figure 5c). Although this interaction occurs through Q191's backbone amine, even small perturbations of the backbone entailed by other side chains may be enough to critically impair folding and/or NADP binding. It is also noteworthy that Q191 is part of the NADP domain, but connects to the BVMO's so‐called helical domain through a hydrogen bond of the amide with S214. The residue thus might also be critical for domain orientation and/or movement, which may occur in these proteins during catalysis (Fürst, Fiorentini, & Fraaije, 2019). Performing a multiple sequence alignment, we also found Q191 to be highly conserved in BVMOs, further suggesting an important functional role (Figure S11).

Interestingly the final stabilized mutant contains most mutations in the FAD domain (Figure 5d). While some mutations were found in the NADP domain in the single mutant screen, they proved incompatible in the combination mutants. Also one of the two mutations in the helical domain was discarded in this step.

The fact that we found a thermostability plateau in the combinations of the first single mutant set let us to believe that we were missing out on stabilizing an early‐unfolding region. If such a region triggers irreversible unfolding at a fixed temperature, this could mask a stabilization towards higher temperatures in another region. This hypothesis is also substantiated by the fact that the mutations of Set 2 gave a more than additive effect. Possibly, these mutations prevented early‐unfolding, thereby unmasking the less than additive effects observed for the previous mutants.

## 4 CONCLUSIONS

CHMO has been a frequent target of protein engineering, despite its complex catalytic mechanism: to overcome the challenge of coordinating oxygen, two cofactors, and the organic substrate, these enzymes employ protein domain and loop movements as well as cofactor rearrangement (Fürst et al., 2019). This hampers many computational design approaches including the here applied FRESCO predictions, as molecular dynamics simulations are unable to cover the timescales of large protein motions. As FRESCO's core algorithms are restricted to the proteinogenic component of the enzyme, the cofactor‐dependency was an additional limitation and required us to exclude approximately 19% of the enzyme's residues from the calculations. Our subsequent screening confirmed the reliability of those predictions, however, with nearly half of the tested mutations exhibiting a stabilizing effect. An innovative shuffled library design method then aided in identifying the most stable mutant. The stabilizing effects of the here‐identified mutations were context‐dependent and we found that the full potential of initially non‐additive mutations can emerge after stabilizing other protein regions. With 49°C, our best mutant shows a slightly higher *T*_m_ than a recently discovered naturally thermotolerant CHMO (Romero et al., 2016) while performing catalysis at a roughly 2.5x higher maximum rate. Our mutant also shows a vastly extended lifetime, and our results confirm previous findings that *T*_m_ and half‐life are strongly correlated (Figure 4c). Our observations suggest a proportional dependency of the two parameters when considering variants of the same enzyme, but not necessarily when comparing different enzymes. In analogy, comparison with literature is straightforward for *T*_m_ values, but difficult for long‐term stability data. Although such comparisons have been made (Balke et al., 2018), the vast discrepancy across studies is exemplified in the literature data on wild‐type AcCHMO stability spanning three orders of magnitude (Goncalves et al., 2017). Until a single study reproducibly compares all available variants under the same conditions, we believe that *T*_m_ values obtained by fluorescent shift assays remain the most reliable and reproducible constant for a first estimate. Total turnover numbers are also better comparable in principal; however, they heavily depend on the reaction conditions, whose optimization for a particular system is laborious as it requires the careful adjustment of many parameters at once (Engler, Gruetzner, Kandzia, & Marillonnet, 2009). As long as conditions are constant within a single study, relative improvements, such as the here presented *T*_m_ shift of 13°C and 30x extended half‐life are potentially the most robust measure.

Whether or not this improvement is sufficient for application remains to be seen. Even wild‐type AcCHMO has already been used in industry (Doig et al., 2002), showing that the particular process economics are not only dictated by enzyme stability, but also for example on specific activity, substrate loading, downstream processing and product price (Tufvesson, Lima‐Ramos, Nordblad, & Woodley, 2011). Thus, stabilized variants like the one presented here contribute to shift biocatalysis from a niche technology to broader application.

## Supporting information

Supplementary information
