# Supplementary material for: Stabilization of cyclohexanone monooxygenase by computational and experimental library design
Source: Biotechnol Bioeng. 2019 Jun 24;116(9):2167–77. doi: 10.1002/bit.27022 (PMC6836875; doi:10.1002/bit.27022)
Supplement: Supplementary file 1 — Supplementary information [file BIT-116-2167-s001.docx]

Supplementary Information

Stabilization of cyclohexanone monooxygenase by computational and experimental library design

Maximilian J. L. J. Fürst^a^, Marjon Boonstra^a^, Selle Bandstra^a^, and Marco W. Fraaije^a^*

^a^Molecular Enzymology Group, University of Groningen, Nijenborgh 4, 9747AG, Groningen, The Netherlands

Short title: Stabilization of cyclohexanone monooxygenase

*Corresponding author: Prof. Dr. Marco W. Fraaije

Molecular Enzymology Group

University of Groningen

Nijenborgh 4

9747AG, Groningen

The Netherlands

E-mail: [m.w.fraaije@rug.nl](mailto:m.w.fraaije@rug.nl)

Tel.: +31 50 36 34345

**Contents**

[Supplementary Figures S2](#_Toc951672)

[Figure S1. Overview of the individual steps followed in the RhCHMO stabilization work flow. S2](#_Toc951673)

[Figure S2. Correlation between the Tm of RhCHMO mutants determined in cell-free extract preparations or as purified proteins. S3](#_Toc951674)

[Figure S3. First approach to generate a shuffled library. S3](#_Toc951675)

[Figure S4. Distribution of wild-type and mutant residues at the targeted positions in the first library. S4](#_Toc951676)

[Figure S6. Electropherograms obtained from sequencing a mixed culture inoculated with all 48 clones of the second library. S4](#_Toc951677)

[Figure S7. Enzyme kinetics. S5](#_Toc951678)

[Figure S8. Activity over time upon incubation at 37 °C. S5](#_Toc951679)

[Figure S9. MD simulation trajectories for wild-type (A) and Q409P mutant (B). S5](#_Toc951680)

[Figure S10. Molecular surfaces of RhCHMO wild type (A) and mutant (B), colored by electrostatic potential distribution. S6](#_Toc951681)

[Figure S11. Weblogo showing sequence conservation among 79 BVMO sequences. S6](#_Toc951682)

[Supplementary Tables S7](#_Toc951683)

[Table S1. S7](#_Toc951684)

References S7

# Supplementary Figures


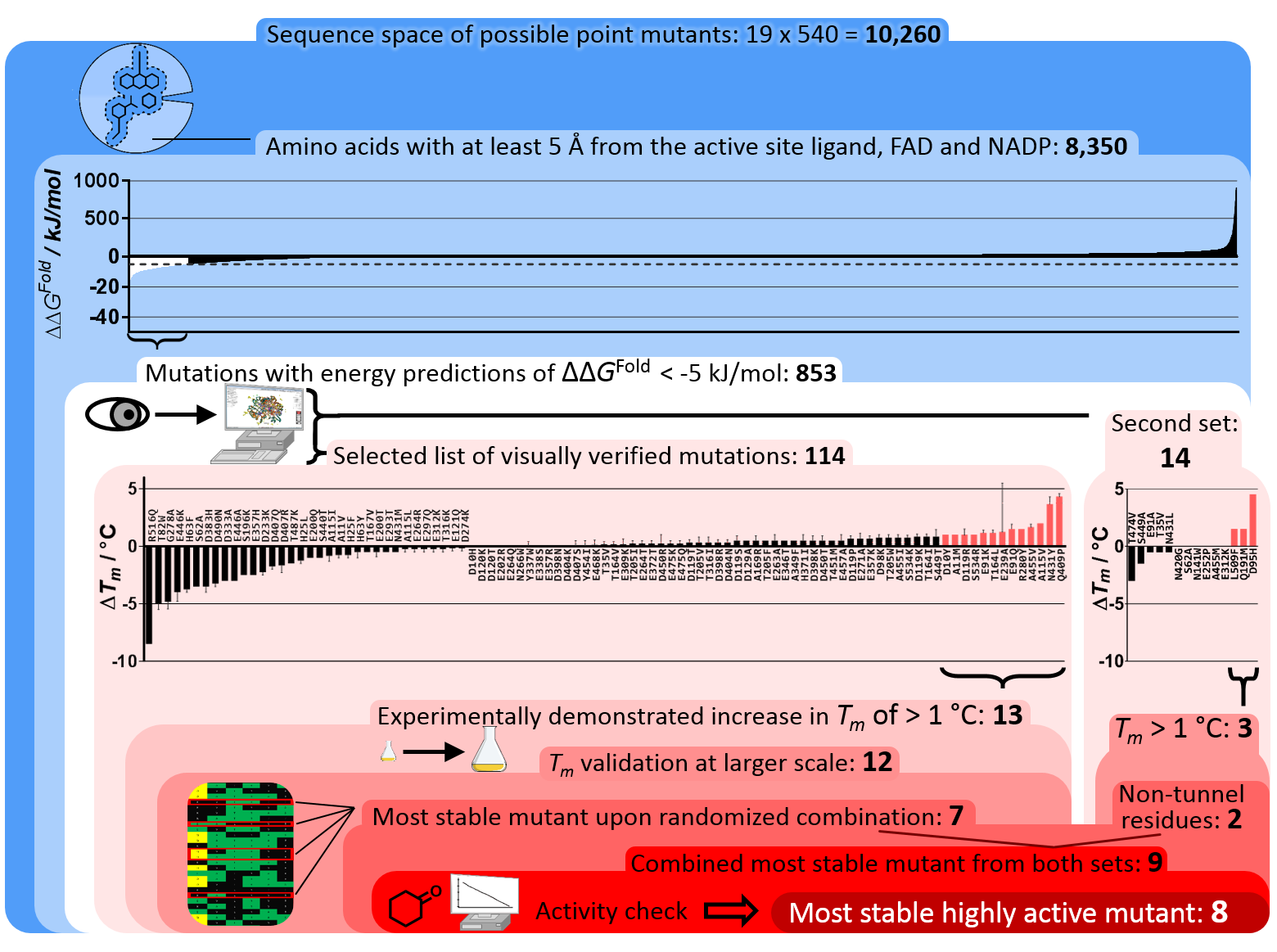


## **Figure S1. Overview of the individual steps followed in the RhCHMO stabilization work flow.** The ΔΔG^Fold^ energy predictions were performed on 8,350 single mutants, of which 853 fell below the energy cutoff of -5 kJ ^mol-1^ (white bars in the top graph). 114 point mutations were intially selected in the visual inspection screen, to which another 14 were added as a second set. The best mutants were combined, partly by using a shuffled library screening approach. The best mutant was obtained after removing one activity-abolishing mutation from the most stable mutant.


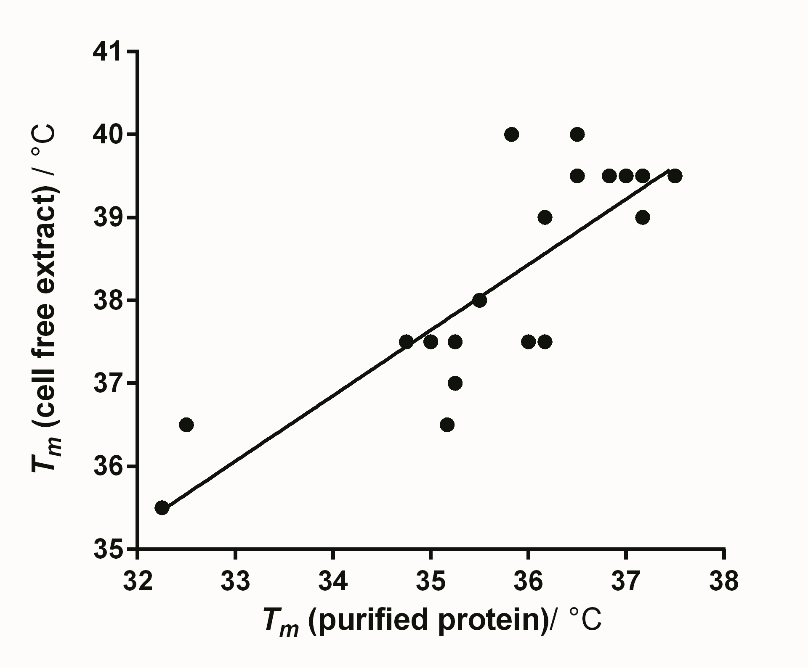


## **Figure S2. Correlation between the *T_m_* of RhCHMO mutants determined in cell-free extract preparations or as purified proteins.** As the single mutant screen requires a certain degree of accuracy, the observed varations between the two enzyme formulations were deemed to large to allow *T_m_* measurements in cell-free extracts.


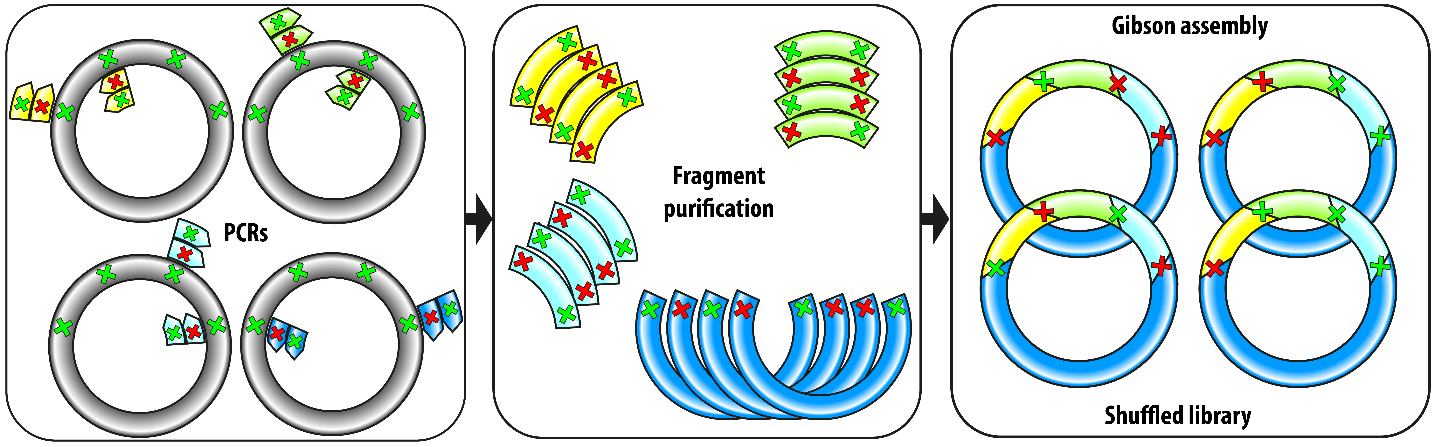


## **Figure S3. First approach to generate a shuffled library.** The method is an adaptation of the so-called multichange isothermal (MISO) mutagenesis procedure (Mitchell et al., 2013). The PCR was performed using a mix of mutated and wild-type primers, after which the mixed fragments were purified and assembled by Gibson cloning (Gibson, 2011).


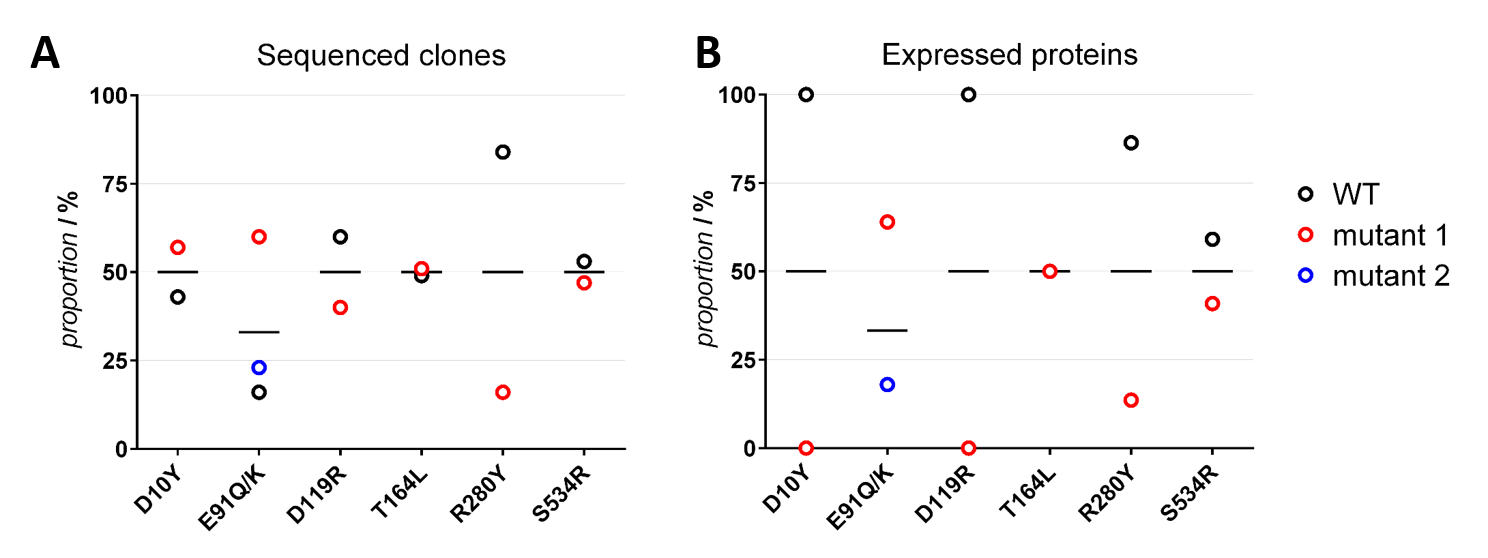


## Figure S4. Distribution of wild-type and mutant residues at the targeted positions in the first library. A) Distribution on sequence level of 95 analyzed clones. B) Distribution in the ThermoFAD signals, resulting from only from mutants that express solubly and bind FAD.


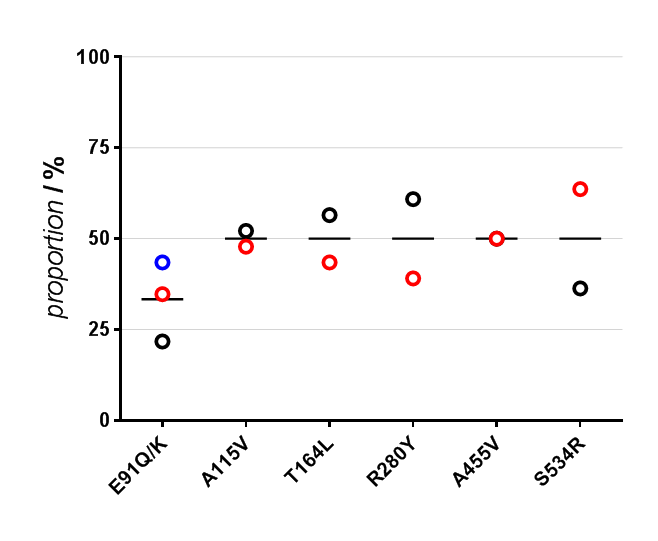


**Figure S5. Distribution of wild-type and mutant residues at the targeted positions in the second library on sequence level of 48 analyzed clones.**


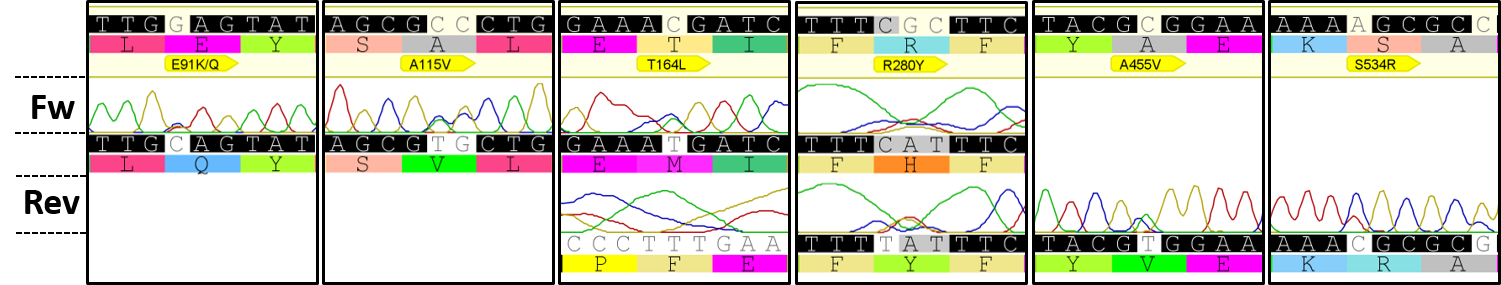


**Figure S6. Electropherograms obtained from sequencing a mixed culture inoculated with all 48 clones of the second library.** Targeted positions show a mixed signal corresponding to the distribution observed when sequencing the clones individually (Figure S5).

##
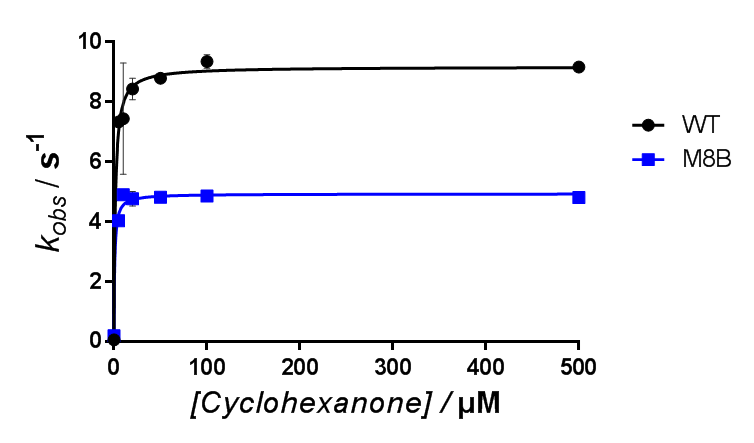
 Figure S7. Enzyme kinetics. Catalytic rates observed upon incubation of RhCHMO wild type (WT) or M8B mutant with varying amount of substrate fitted to the Michaelis-Menten equation.


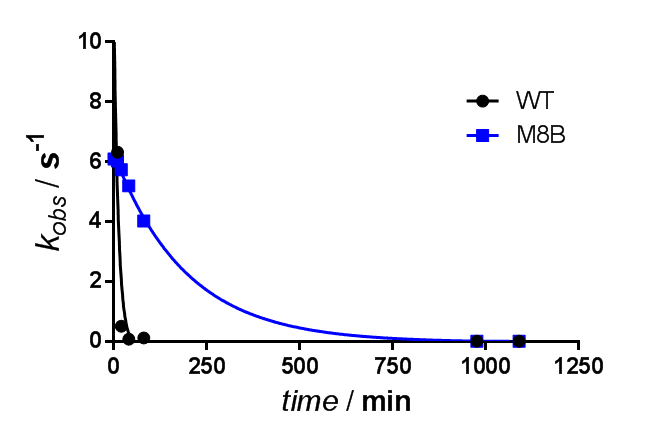


## Figure S8. Activity over time upon incubation at 37 °C.


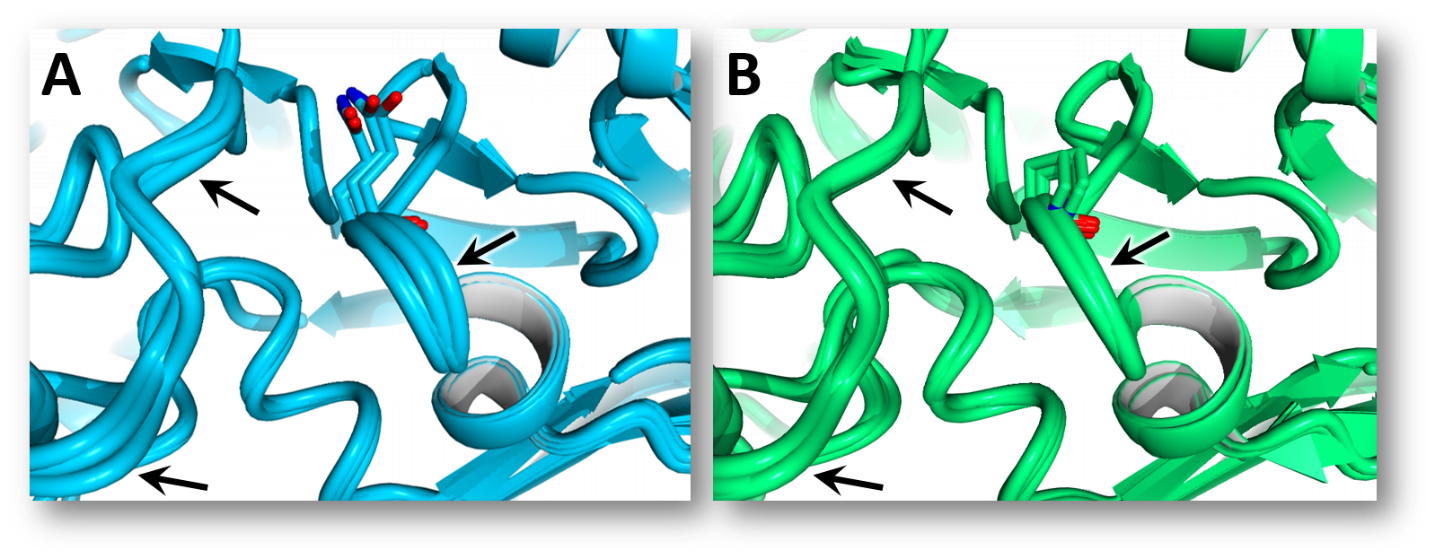


## Figure S9. MD simulation trajectories for wild-type (A) and Q409P mutant (B). The average structure of four independent trajectories is overlaid and shows more deviation (and thus more flexibility) in the with arrow indicated positions for the wild type than for the mutant.


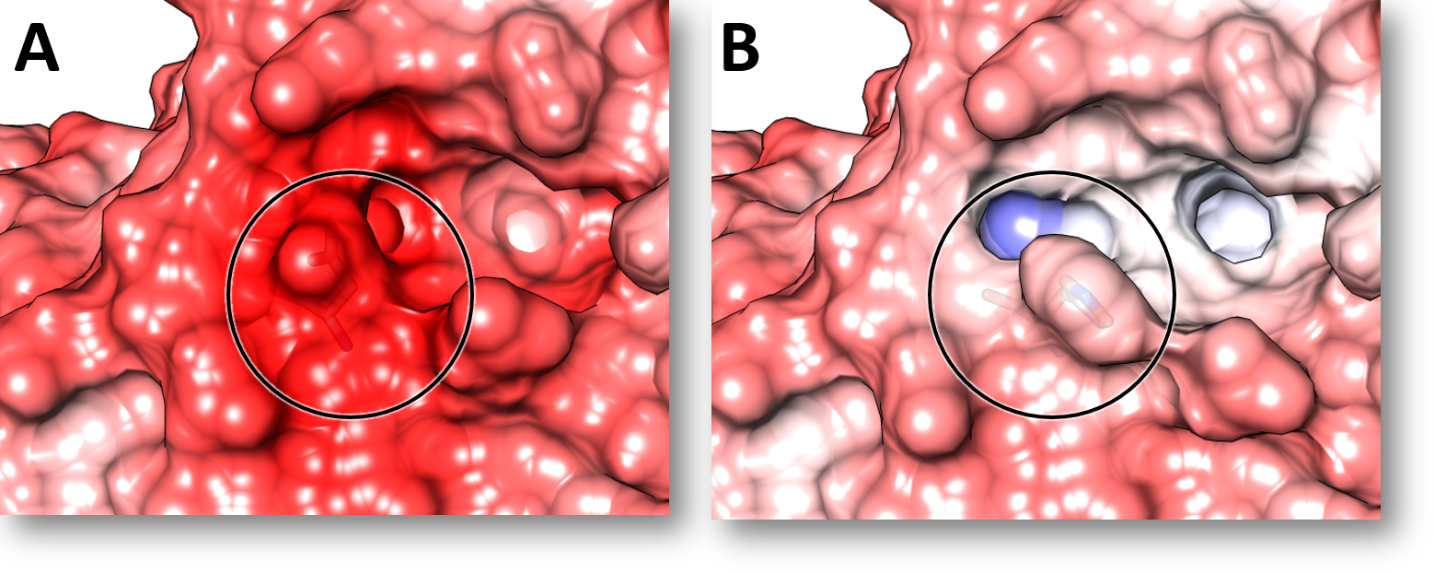


## Figure S10. Molecular surfaces of RhCHMO wild type (A) and D95H mutant (B), colored by electrostatic potential distribution. Negative charge is shown red, neutral is white, and positive blue. The potentials were calculated using the adaptive Poisson–Boltzmann Solver (APBS) web server (Jurrus et al., 2018).


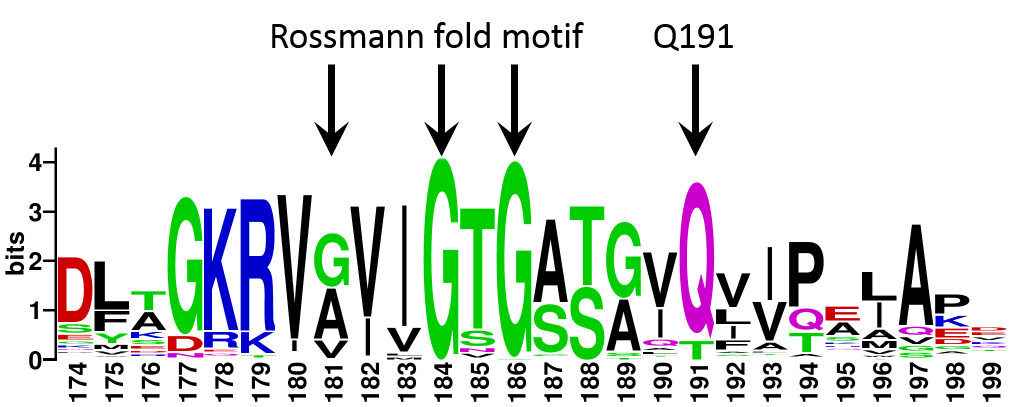


## Figure S11. Weblogo showing sequence conservation among 79 BVMO sequences. The Rossmann fold motif GXXGXG and the mutated Q191 are marked. The sequences are a set of literature-described BVMOs that were recombinantly expressed. The full list of sequences and their accession numbers are documented in Figure S1 of Fürst et al., 2017.

# Supplementary Tables

## Table S1. P-values obtained from a χ^2^-test on the observed wild type/mutant distribution (Figure S5).

| Mutation | P-value^a^ |
| --- | --- |
| E91/QK | 0.191633 |
| A115V | 0.768083 |
| T164L | 0.376344 |
| R280Y | 0.140369 |
| A455V | 1 |
| S534R | 0.07044 |

^a^values < 0.005 signify a statistically significant difference between the two values.

# References

Fürst, M. J. L. J., Savino, S., Dudek, H. M., Gomez Castellanos, J. R., Gutierrez de Souza, C., Rovida, S., . . . Mattevi, A. (2017). Polycyclic Ketone Monooxygenase from the Thermophilic Fungus Thermothelomyces thermophila: A Structurally Distinct Biocatalyst for Bulky Substrates. *Journal of the American Chemical Society, 139*(2), 627-630. <https://doi.org/10.1021/jacs.6b12246>

Gibson, D. G. (2011). Enzymatic assembly of overlapping DNA fragments. In C. Voigt (Ed.), *Methods in Enzymology* (Vol. 498, pp. 349-361). New York, NY: Academic Press. <https://doi.org/https://doi.org/10.1016/B978-0-12-385120-8.00015-2>

Jurrus, E., Engel, D., Star, K., Monson, K., Brandi, J., Felberg, L. E., . . . Liles, K. (2018). Improvements to the APBS biomolecular solvation software suite. *Protein Science, 27*(1), 112-128. <https://doi.org/https://doi.org/10.1002/pro.3280>

Mitchell, L. A., Cai, Y., Taylor, M., Noronha, A. M., Chuang, J., Dai, L., & Boeke, J. D. (2013). Multichange Isothermal Mutagenesis: A New Strategy for Multiple Site-Directed Mutations in Plasmid DNA. *ACS Synthetic Biology, 2*(8), 473-477. <https://doi.org/10.1021/sb300131w>
